# Concealing the Taste of Fish Oil

**Published:** 1849-12

**Authors:** 


*Concealing the Taste of Fish Oil.*—Now that the swallowing cod-liver oil bids fair to become the fashionable mania of the day, it may be as well to state the simple and effectual means communicated by M. Fredericq, of disguising its abominable taste. This merely consists in masticating a morsel of dried orange peel just before and just after swallowing the dose. —*Rev. Med. Chir.*, tom. v.

[We have employed this means in one case, and with a very satisfactory result. The patient states that the taste of the oil is entirely disguised by the use of the orange peel.]

